# Modelling lung diffusion-perfusion limitation in mechanically ventilated SARS-CoV-2 patients

**DOI:** 10.3389/fphys.2024.1408531

**Published:** 2024-07-12

**Authors:** Giuseppe Miserocchi, Emanuele Rezoagli, Agueda Muñoz-Del-Carpio-Toia, Leydi Pamela Paricahua-Yucra, Natalia Zubieta-DeUrioste, Gustavo Zubieta-Calleja, Egidio Beretta

**Affiliations:** ^1^ Dipartimento di Medicina e Chirurgia, Università Milano-Bicocca, Monza, Italy; ^2^ Department of Emergency and Intensive Care, Fondazione IRCCS San Gerardo dei Tintori, Monza, Italy; ^3^ Vicerrectorado de Investigación, Escuela de Postgrado Universidad Católica de Santa María, Arequipa, Peru; ^4^ Unidad de Cuidados Intensivos, Hospital Nacional Carlos Alberto Seguin Escobedo, Arequipa, Peru; ^5^ High Altitude Pulmonary and Pathology Institute (HAPPI-IPPA), La Paz, Bolivia

**Keywords:** dead space, respiratory compliance, gas exchanges, diffusion limitation, perfusion limitation, alveolar pressure, lung distension, mechanical ventilation

## Abstract

This is the first study to describe the daytime evolution of respiratory parameters in mechanically ventilated COVID-19 patients. The data base refers to patients hospitalised in the intensive care unit (ICU) at Arequipa Hospital (Peru, 2335 m) in 2021. In both survivors (S) and non-survivors (NS) patients, a remarkable decrease in respiratory compliance was observed, revealing a proportional decrease in inflatable alveolar units. The S and NS patients were all hyperventilated and their SatO_2_ was maintained at >90%. However, while S remained normocapnic, NS developed progressive hypercapnia. We compared the efficiency of O_2_ uptake and CO_2_ removal in the air blood barrier relying on a model allowing to partition between diffusion and perfusion limitations to gas exchange. The decrease in O_2_ uptake was interpreted as diffusion limitation, while the impairment in CO_2_ removal was modelled by progressive perfusion limitation. The latter correlated with the increase in positive end-expiratory pressure (PEEP) and plateau pressure (Pplat), leading to capillary compression, increased blood velocity, and considerable shortening of the air-blood contact time.

## 1 Introduction

Respiratory failure can develop when lung disease forces a patient to unsuccessfully adapt his ventilatory response to ensure gas exchange. The management of severe lung diseases involving loss of function in the alveolar units remains a challenge in intensive care units. From a pathophysiological point of view, the critical issue is that the spreading of lung disease can lead to the progressive loss of specific morpho-functional features of the air-blood barrier that normally ensure gas diffusion (Rezoagli et al., 2022). The efficiency of gas exchange is based not only on the morphological integrity of the alveolar-capillary membrane but also on the functional coupling between the gas diffusion capacity and perfusion capacity. The importance of this coupling has recently been emphasised, providing additional information on facing a perturbation in gas exchange (Beretta et al., 2019; Miserocchi et al., 2022; Miserocchi, 2023a). Based on our past work on the air blood barrier function, we have been invited to comment on a database of daytime evolution of respiratory parameters in mechanically ventilated SARS-CoV-2 patients. SARS-CoV-2 respiratory failure has led to a massive need for mechanical ventilatory support worldwide (Rezoagli et al., 2021). This study wishes to explore the impact of SARS-CoV-2 disease on the diffusion/perfusion function in mechanical ventilated patients.

## 2 Material and methods

This was a retrospective study based on data from adult COVID patients hospitalised in the intensive care unit (ICU) at Arequipa Hospital (Peru, 2335 m) in 2021. The study was conducted in accordance with the Declaration of Helsinki. Patient consent was waived owing to the observational nature of the study, and the Institutional Review Board of Arequipa Hospital approved the data collection. No patient identifiers were used in this study.

Patients with a clinical diagnosis of respiratory failure and with a positive confirmation at the PCR quantification of Sars-CoV2 infection by sample evaluation from airways (i.e., naso-pharingeal swabs, bronchoaspirate, bronchoalveolar lavage) were intubated according to Institution standard of care of the admitting Intensive Care Unit and were enrolled in the current analysis. No specific exclusion criteria were considered. Settings of mechanical ventilation were applied in accordance to the recommendations of protective mechanical ventilation as reported in the ARDS guidelines (Fan et al., 2017).

All patients were maintained in the supine position, 30° head up (Spooner et al., 2014). The following parameters were collected daily during mechanical ventilation in ICU: tidal volume (Vt), respiratory rate (RR), minute ventilation ( $\dot{V}E=Vt\cdot RR$ ) normalized to body weight ( $\dot{V}E$ /kg), positive end-expiratory pressure (PEEP), plateau pressure (Pplat), driving pressure (DP) calculated as Pplat-PEEP, FIO_2_, SatO_2_, arterial PaO_2_, and PaCO_2_. Reference values for PaO_2_ and PaCO_2_ at 2335 m are approximately 75 mmHg and 32.5 mmHg, respectively (Ramirez-Sandoval et al., 2016). P/F was calculated as the PaO_2_ over FIO_2_ ratio. Respiratory system compliance (Crs, ml · cmH_2_O^−1^) was calculated as the ratio Vt/(Pplat-PEEP). We derive %lung distension at PEEP and Pplat from the average Pressure-Volume curve of the respiratory system (Agostoni and Mead, 1964) and Ventilatory ratio ( ${\dot{V}}_{R}$ ) defined as ${\dot{V}}_{R}=\frac{{{\dot{V}}_{E}}_{measured\;}\cdot {{PaCO}_{2}}_{measured}}{{{\dot{V}}_{E}}_{predicted\;}\cdot {{PaCO}_{2}}_{ideal}}$ where ${{\dot{V}}_{E}}_{predicted\;}$ is calculated as body weight · 100 mL/min and ${{PaCO}_{2}}_{ideal}$ is set at 37.5 mmHg (Sinha et al., 2013).

Statistics: descriptive data were reported as mean ± standard deviation; differences between continuous variables were reported as t-tests paired and unpaired as appropriate; correlations of continuous data were assessed by Pearson’s correlation coefficient; alpha level<0.05 was deemed significant (two-tailed). All statistical analyses were performed using Microsoft Excel (Version 16.81).

## 3 Results

Table 1 reports data for survivors (S, n = 30; 26 males, 87%) and non-survivors (NS, n = 16; 15 males, 94%) patients referring to the first day of admission in ICU.

**TABLE 1:** Data for survivors (S) and non-survivors (NS) patients on the first day of admission in ICU.

Survivors															
n°	Weight	Crs	PEEP	Vt	%Lung dist at PEEP	Pplat	% Lung dist at Pplat	FIO_2_	%SatO_2_	PaO_2_	PaCO_2_	P/F	$\dot{V}E$	DP	VR
1	61.4	25	8	418	44	25	82	0.5	98	95	41	190	177.0	17	2.0
2	65.7	51	10	482	50	20	72	0.6	96	84	40	141	183.5	10	1.9
3	66.0	43	12	474	55	23	79	0.7	96	82	43	117	186.7	11	2.1
4	70.6	36	11	499	53	25	82	0.7	95	94	33	144	176.7	14	1.5
5	71.5	32	12	473	55	27	85	0.7	95	92	33	131	165.4	15	1.5
6	66.0	32	16	441	65	30	90	0.9	93	75	36	84	187.1	14	1.8
7	68.7	21	10	480	50	23	79	0.7	96	85	23	121	209.7	13	1.3
8	71.5	30	12	603	55	32	92	0.7	97	65	20	93	253.1	20	1.3
9	72.0	35	14	563	60	30	90	0.8	94	98	36	123	218.9	16	2.1
10	64.0	23	18	384	69	35	95	1.0	95	81	41	81	180.0	17	2.0
11	70.5	30	9	502	47	26	84	0.5	96	75	37	150	206.5	17	2.0
12	81.0	32	14	442	60	28	87	0.7	97	123	38	176	158.2	14	1.6
13	51.5	23	10	418	50	28	87	0.8	97	73	31	92	243.5	18	2.0
14	41.5	10	10	291	50	38	98	0.5	94	70	43	140	245.4	28	2.8
15	63.3	19	14	356	60	33	93	0.9	95	79	57	88	180.0	19	2.7
16	63.3	21	12	246	55	24	81	0.7	97	134	26	191	124.4	12	0.9
17	65.0	34	13	387	57	24	81	0.8	99	158	48	205	131.0	11	1.7
18	63.0	24	14	307	60	27	85	0.7	96	63	77	90	160.8	13	3.3
19	66.0	44	13	394	58	22	77	0.8	98	87	50	108	137.3	9	1.8
20	69.7	27	10	426	50	26	84	0.5	97	99	48	198	158.9	16	2.0
21	66.0	30	10	446	51	25	82	0.6	96	50	37	79	164.1	15	1.6
22	48.8	32	14	351	60	25	82	0.8	95	77	36	96	201.1	11	1.9
**23**	**73.2**	**45**	**14**	446	**60**	**24**	**81**	**0.9**	**93**	**83**	**22**	**92**	**158.5**	10	0.9
24	60.6	26	14	342	60	27	86	0.8	95	71	46	88	158.0	13	1.9
25	67.8	27	10	439	50	26	84	0.6	98	119	42	198	181.3	16	2.0
26	61.5	27	16	439	65	32	92	0.9	95	72	44	80	249.8	16	2.9
27	61.5	18	16	246	65	30	90	1.0	94	82	45	82	140.0	14	1.7
28	71.2	43	14	445	59	24	81	0.8	98	109	27	136	175.0	10	1.3
29	61.5	49	14	417	60	23	78	0.7	95	66	40	95	176.3	9	1.9
30	65.1	38	14	472	60	27	85	0.8	94	81	32	103	217.5	13	1.8
**mean**	**65**	**31**	**13**	**421**	**56**	**27**	**85**	**1**	**96**	**87**	**39**	**124**	**184**	**14**	**2**
**SD**	**7.6**	**10**	**2**	**81.9**	**5.9**	**4**	**5.8**	**0.1**	**2**	**23**	**11**	**41**	**35**	**3.9**	**0.5**

NON-SURVIVORS															
n°	Weight	Crs	PEEP	Vt	%Lung dist at PEEP	Pplat	% Lung dist at Pplat	FIO_2_	%SatO_2_	PaO_2_	PaCO_2_	P/F	$\dot{V}E$	DP	VR
31	64.1	37	14	484	60	27	85	0.8	87	52	33	65	226.5	13	2.0
32	60.9	52	14	413	60	22	77	0.9	95	99	39	116	203.4	8	2.1
33	78.0	34	12	475	55	26	84	0.8	94	76	37	95	170.5	14	1.7
34	73.2	43	15	478	62	26	84	0.9	95	78	38	87	195.9	11	2.0
35	61.4	21	16	354	65	33	93	1.0	96	72	45	76	190.3	17	2.3
36	58.7	37	12	447	55	24	81	0.7	95	174	34	249	213.1	12	1.9
37	61.0	14	11	315	52	33	93	1.0	86	56	35	56	185.9	22	1.7
38	61.0	20	14	377	60	33	93	0.9	90	58	40	64	185.4	19	2.0
39	64.0	19	12	368	55	31	91	0.7	94	82	32	118	149.5	19	1.3
40	63.0	22	14	381	60	31	91	0.9	96	84	45	93	181.4	17	2.2
41	79.0	19	14	336	60	32	92	1.0	96	81	80	81	153.1	18	3.3
**42**	**42.5**	**25**	**13**	272	**58**	**24**	**81**	**0.7**	**94**	**73**	**27**	**99**	**202.2**	11	1.5
43	61.5	36	14	364	60	24	81	0.9	93	85	62	94	171.6	10	2.8
44	52.4	14	16	296	65	37	97	1.0	88	42	31	42	158.1	21	1.3
45	43.3	35	13	347	58	23	79	0.7	96	74	37	114	224.4	10	2.2
46	57.8	25	14	361	60	29	88	0.7	99	89	32	127	187.4	15	1.6
**mean**	**61.4**	**28**	**14**	**379**	**59**	**28**	**87**	**0.8**	**93**	**80**	**40**	**99**	**187**	**15**	**2.0**
**SD**	**10.1**	**11.1**	**1.4**	64.5	**3.5**	**4.5**	**6.3**	**0.1**	**3.7**	**29.2**	**13.3**	**46.6**	**23.3**	**4.3**	**0.5**

Patients #23 and #42 (in bold) were selected as representative patients of the survivors (S) and non-survivors (NS) groups.

Respiratory compliance (Crs) is expressed in ml·cmH_2_O^−1^, Tidal Volume (Vt) in ml, minute ventilation ( $\dot{V}E$ ) in ml·kg^−1^·min^−1^; Positive End-Expiratory Pressure (PEEP), Plateau pressure (Pplat), and driving pressure (DP) are expressed in cmH_2_O; PEEP %lung distension and Pplat %lung distension are expressed as percent of vital capacity from the standard Pressure-Volume curve of the respiratory system (Agostoni and Mead, 1964); PaO_2_, PaCO_2_, and P/F are expressed in mmHg; FIO_2_, and Ventilatory Ratio (VR) are pure numbers; %SatO_2_ percent of arterial oxygen saturation.

In bold, mean values ± Standard Deviation. t-test unpaired: no significant differences were found in comparing S vs. NS.

Table 2 reports data for S and NS patients referring to the last day of ICU.

**TABLE 2:** Data for survivors (S) and non-survivors (NS) patients on the last day in ICU.

Survivors															
n°	Days in ICU	Crs	Vt	PEEP	%Lung dist at PEEP	Pplat	% Lung dist at Pplat	FIO_2_	%SatO_2_	PaO_2_	PaCO_2_	P/F	$\dot{V}E$	DP	VR
1	21	40	477	5	36	17	67	0.3	96	75	30	251	209.8	12	1.7
2	17	73	433	5	36	13	58	0.3	99	75	31	187	197.8	8	1.7
3	22	46	555	5	36	17	67	0.3	96	80	30	267	143.0	12	1.1
4	21	30	548	5	36	23	79	0.4	95	79	38	198	186.3	18	1.9
5	29	33	394	5	36	17	67	0.4	99	72	32	180	165.3	12	1.4
6	21	26	475	5	36	23	79	0.5	96	86	37	173	237.5	18	2.3
7	4	34	478	8	44	22	77	0.6	95	77	30	140	167.0	14	1.3
8	9	20	378	5	36	24	81	0.5	96	67	31	135	169.2	19	1.4
9	17	33	522	8	44	24	81	0.5	93	84	33	168	181.3	16	1.6
10	43	19	442	5	36	28	87	0.4	94	85	37	212	241.7	23	2.4
11	4	78	547	5	36	12	55	0.4	97	95	36	238	170.7	7	1.6
12	13	40	495	5	36	21	75	0.3	97	69	27	230	171.1	16	1.2
13	33	19	514	5	36	32	92	0.4	96	66	45	164	269.5	27	3.2
14	11	16	257	5	36	23	79	0.4	95	72	22	180	167.2	18	1.0
15	38	15	460	5	36	36	96	0.4	96	90	45	225	181.7	31	2.2
16	4	21	288	5	36	19	71	0.4	94	87	24	218	81.9	14	0.5
17	5	40	554	5	36	19	71	0.4	96	85	41	211	204.6	14	2.2
18	9	16	410	5	36	30	90	0.5	95	73	42	146	169.2	25	1.9
19	7	38	574	5	36	20	73	0.5	94	62	31	124	208.7	15	1.7
20	4	32	532	7	40	23	79	0.4	94	76	37	189	160.2	17	1.6
21	50	18	405	5	36	27	85	0.5	96	79	40	157	191.5	22	2.0
22	10	29	401	5	36	19	71	0.4	95	62	38	173	221.6	14	2.2
**23**	**23**	**26**	486	**5**	**36**	**24**	**81**	**0.3**	**96**	**66**	**35**	**218**	**212.5**	**19**	**2.0**
24	12	30	393	5	36	18	69	0.4	96	73	38	169	155.6	13	1.6
25	4	30	507	5	36	22	77	0.4	96	101	30	253	291.6	17	2.3
26	5	131	590	8	43	12	55	0.4	96	72	34	168	193.2	5	1.8
27	17	32	422	5	36	18	69	0.4	96	83	23	208	178.4	13	1.1
28	4	23	475	5	36	26	83	0.4	95	91	33	227	173.5	21	1.5
29	12	46	458	8	44	18	69	0.5	95	70	38	156	178.7	10	1.8
30	20	56	558	5	36	15	62	0.4	95	84	43	209	171.4	10	2.0
**mean**	**16**	**36**	**468** ^**##**^	**5** ^**##**^	**37** ^**##**^	**21** ^**##**^	**75** ^**##**^	**0.4** ^**##**^	**96**	**78** ^**##**^	**34** ^**##**^	**192** ^**##**^	**188**	**16**	**2**
**SD**	**12.3**	**23.5**	80.1	**1.0**	**2.9**	**5.6**	**10.3**	**0.07**	**1.3**	**9.7**	**6.1**	**37.2**	**39.1**	**5.9**	**0.5**

NON-SURVIVORS															
n°	Days in ICU	Crs	Vt	PEEP	%Lung dist at PEEP	Pplat	% Lung dist at Pplat	FIO_2_	%SatO_2_	PaO_2_	PaCO_2_	P/F	$\dot{V}E$	DP	VR
31	26	29	459	14	60	30	90	0.9	90	89	60	99	243.5	16	3.9
32	14	18	410	18	69	41	100	1.0	86	48	64	48	228.9	23	3.9
33	10	34	497	14	60	29	88	0.8	94	67	36	84	184.8	15	1.8
34	31	22	512	14	60	37	97	1.0	91	72	54	72	244.8	23	3.5
35	44	15	311	10	50	31	91	0.9	90	73	69	81	177.3	21	3.3
36	54	18	445	5	36	30	90	0.6	92	59	55	98	242.4	25	3.6
37	44	10	327	8	44	40	100	1.0	89	56	59	56	187.6	32	2.9
38	29	20	426	15	62	36	97	0.9	92	61	32	67	247.0	22	2.1
39	42	14	367	5	36	32	92	0.6	95	71	65	118	160.6	27	2.8
40	15	12	426	12	55	49	100	1.0	89	64	61	64	202.9	37	3.3
41	17	15	382	8	44	34	94	0.6	94	53	58	89	169.2	26	2.6
**42**	**27**	**9**	186	**14**	**60**	**34**	**94**	**1.0**	**75**	**56**	**100**	**56**	**153.2**	**20**	**4.1**
43	17	26	448	16	65	33	93	1.0	91	72	53	72	255.0	17	3.6
44	20	30	312	12	55	22	78	0.9	94	85	50	100	208.3	10	2.8
45	15	23	276	14	60	26	84	0.9	93	70	67	78	223.1	12	4.0
46	8	17	258	11	51	26	84	0.7	92	65	61	98	125.0	16	2.0
**mean**	**26**	**19** ^**##^	**378** ^**^	**12** ^**^	**54** ^**^	**33** ^**#^	**92**	**0.86** ^**^	**90** ^*^	**66** ^**^	**59** ^**##^	**80** ^**^	**203**	**21** ^**##^	**3.1** ^**##^
**SD**	**13.9**	**7.5**	92.0	**3.8**	**9.8**	**6.6**	**6.7**	**0.16**	**4.7**	**10.9**	**15.0**	**19.3**	**39.5**	**7.2**	**0.7**

Patients #23 and #42 (in bold) were selected as representative patients of the survivors (S) and non-survivors (NS) groups.

Respiratory compliance (Crs) is expressed in ml·cmH_2_O^−1^, Tidal Volume (Vt) in ml, minute ventilation ( $\dot{V}E$ ) in ml·kg^−1^·min^−1^; Positive End-Expiratory Pressure (PEEP), Plateau pressure (Pplat), and driving pressure (DP) are expressed in cmH_2_O; PEEP %lung distension and Pplat %lung distension are expressed as percent of vital capacity from the standard Pressure-Volume curve of the respiratory system (Agostoni and Mead, 1964); PaO_2_, PaCO_2_, and P/F are expressed in mmHg; FIO_2_, and Ventilatory Ratio (VR) are pure numbers; %SatO_2_ percent of arterial oxygen saturation.

In bold mean values ± Standard Deviation. t-test paired:*p < 0.05 S vs. NS, **p < 0.01 S vs. NS*. t-test paired: ^#^<0.05 first vs. last day ICU, ^##^ *p* < 0.01 first vs. last day ICU.

Comparing data on the first and last days of ICU stay (Tables 1, 2) in S (n = 30) patients, we found the following:

- No significant change in Crs, but a significant decrease in PEEP and Pplat (with a corresponding decrease in lung distension);
- All subjects were hyperventilated relative to the standard value of 100 mL kg^−1^·min^−1^.
- Significant increase in diffusion/perfusion efficiency of the air–blood barrier for O_2_ (increase in PaO_2_, P/F, and decrease in FIO_2_) and CO_2_ (decrease in PaCO_2_).

The same comparison for NS (n = 16) patients (Tables 1, 2) shows:

- Significant decrease in Crs with no change in PEEP and significant increase in Pplat (increase in lung distension).
- All subjects were hyperventilated.
- No change in diffusion/perfusion efficiency of the air–blood barrier for O_2_ (PaO_2_, P/F, and decrease in FIO_2_), but a considerable reduction in diffusion/perfusion efficiency for CO_2_ elimination (increase in PaCO_2_).

Upon comparing data from the first day between the S and NS patients, no significant differences were found. However, the same comparison for the last day showed significant differences for all parameters considered, except ventilation, revealing a considerable loss of diffusion/perfusion efficiency of the air–blood barrier concerning O_2_ and CO_2_ and greater overdistension of the lung at both PEEP and Pplat.

We decided to discuss two representative patients from the S and NS groups (patient #23 and #42 in Tables 1, 2, respectively). Both patients showed a comparable decrease in Crs, with opposite fates concerning the diffusion/perfusion efficiency of the air-blood barrier for O_2_ and CO_2_.

### 3.1 Respiratory mechanics and alveolar pressure during mechanical ventilation

**FIGURE 1:**
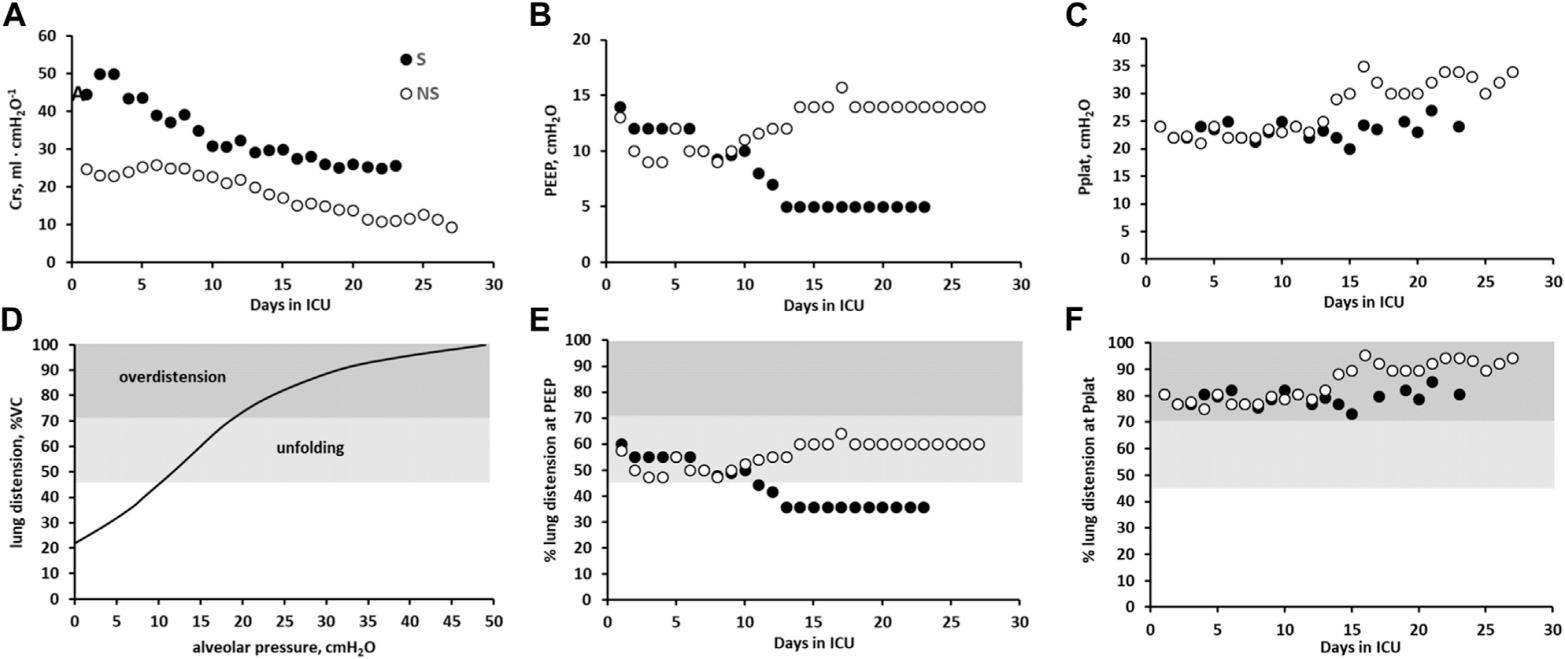
Respiratory parameters in 2 representative patients. Survivors (S, closed circles) and non-survivors (NS, open circles) in all panels. First row. Data referring to the time course of Crs **(A)**, PEEP **(B)**, and Pplat **(C)** Second row: Panel **(D)** reports the degree of lung distension based on the volume-pressure curve of the respiratory system in the supine position. Grey levels refer to the alveolar folding/unfolding and lung overdistension zones. Panels **(E,F)** report %lung distension for the two subjects at PEEP and Pplat, respectively.

The first row in Figures 1A–C shows the time course of Crs, PEEP, and Pplat in two representative patients: survivors (S, closed symbols) and non-survivors (NS, open symbols). In both patients, Crs decreased over time to a similar extent, although the Crs values in NS were lower (Panel A). A clear dissociation is seen in panel B, as PEEP increased in NS while decreasing in S. Panel C reports the Pplat values that increased over time in NS but remained essentially steady in S.

Panel D in the second row of Figure 1 shows the pressure-volume relationship of the respiratory system (Agostoni and Mead, 1964), which is expressed as a percentage of the maximum. The maximum volume decreased with decreasing Crs, reflecting a decrease in inflatable alveolar units (IAU). If the mechanical properties of the residual IAU remained unchanged, the curve in Figure 1D reflects the *specific* compliance of the IAU. Based on this assumption, the ordinate can be used to express the corresponding degree of lung distension of the IAU as a function of the alveolar pressure. The light grey area includes the portion of the pressure-volume curve with the highest specific compliance, extending from 45% to 70% lung distension, corresponding to a range of alveolar pressures from approximately 10–20 cmH_2_O. In this range of pressures, the process of unfolding of the alveolar surface takes place on inspiration, reflecting the existence of a “*reserve*” surface area of the corrugated alveolar cells (Weibel, 2015). As the unfolding process develops, the parenchymal stretch gradually increases (from the light to the darker grey area), indicating lung overdistension. Under physiological conditions at rest, an increase in tidal volume during spontaneous breathing is achieved by an increase in transpulmonary pressure of approximately 5 cmH_2_O; accordingly, the same tidal volume in mechanical ventilation would be achieved by an alveolar pressure of approximately 5 cmH_2_O, corresponding to 30% lung distension, well below the saturation of the unfolding zone. Panels E and F show the degree of lung distention at PEEP and Pplat, respectively, for the two subjects. In the case of NS, lung distension at PEEP falls in the light grey area, whereas at Pplat, lung distension falls in the overdistension zone for both patients.

### 3.2 Gas exchange

**FIGURE 2:**
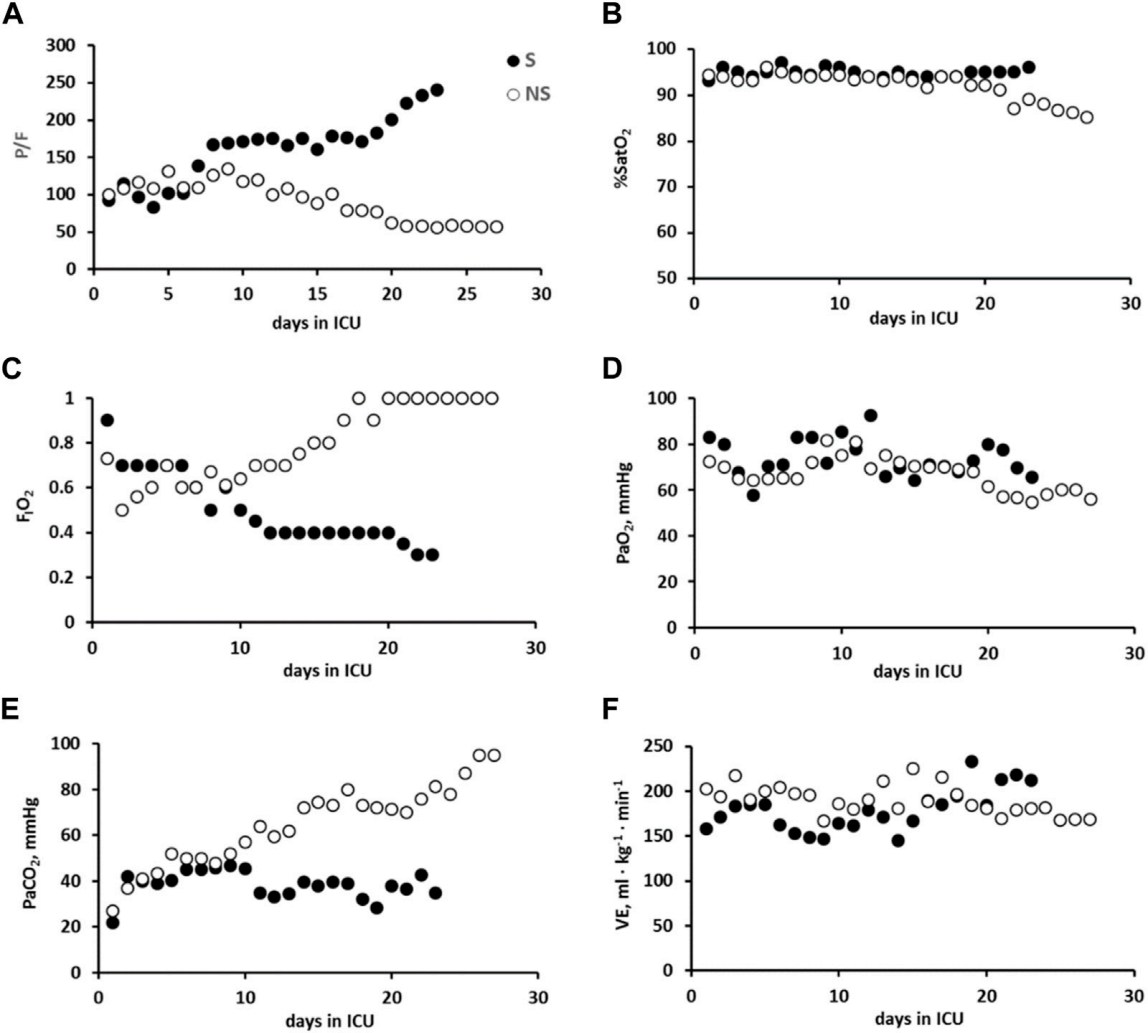
Data referring to the time course of gas exchange in the two representative patients: survivors, S (closed symbols), and non-survivors (NS, open symbols) in all panels.: P/F **(A)**, SatO_2_ **(B)**, FIO_2_ **(C)**, PaO_2_ **(D)**, PaCO_2_ **(E)**, and VE/kg **(F)**.

Panel A in Figure 2 shows that the time course of P/F significantly decreased in both patients. As shown in Panel B, no significant differences in SatO_2_ were observed. Panels C and E again show a divergence in the time courses of FIO_2_ and PaCO_2_, despite displaying a similar time course for PaO_2_ and VE/kg (Panels D and F, respectively).

**FIGURE 3:**
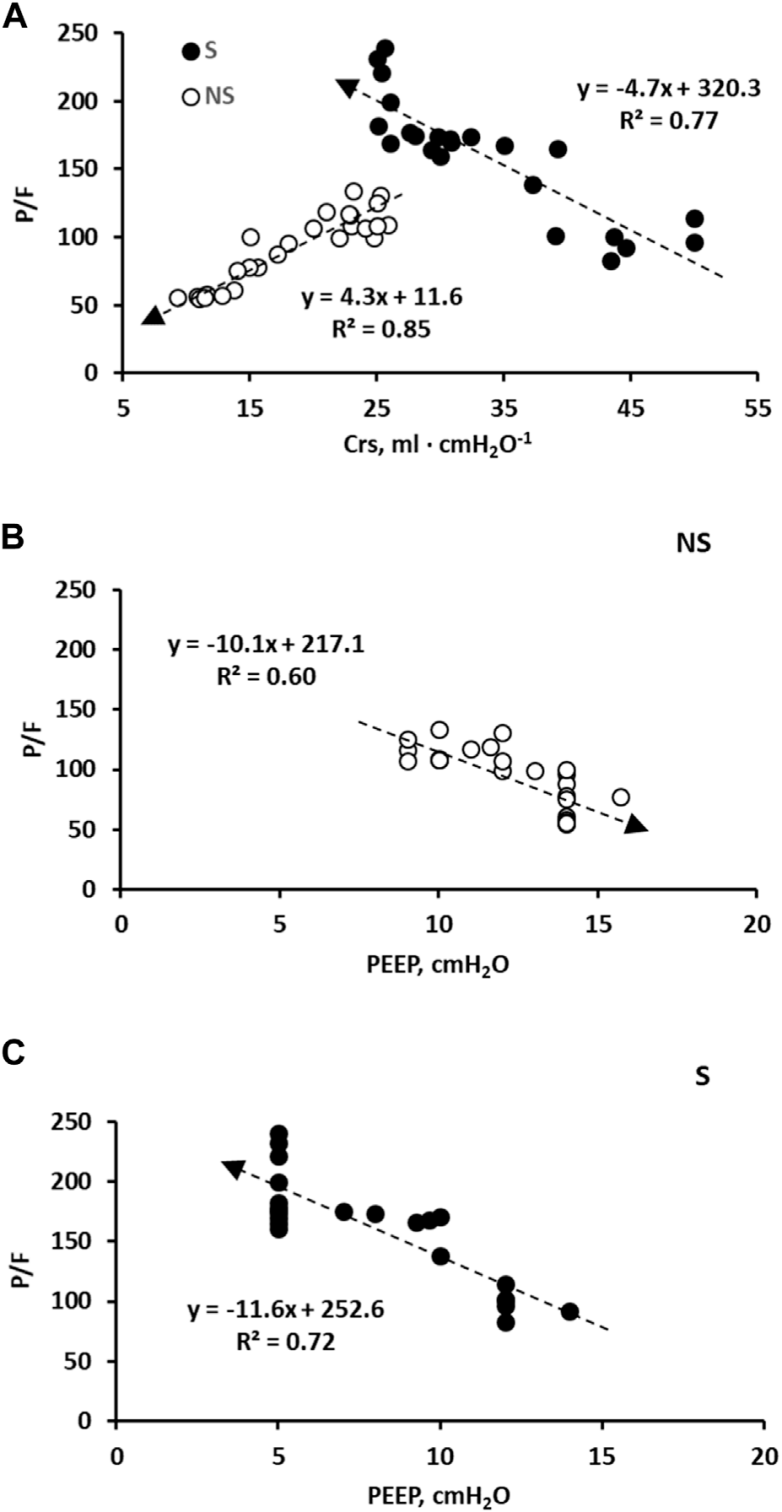
**(A)**: correlation between day-time P/F vs. Crs in the two subjects S (closed symbols) and NS (open symbols). **(B)**: correlation between day-time P/F vs. PEEP in subject NS. **(C)**: correlation between day-time P/F vs. PEEP in subject S. Time dependence is expressed by the arrows in all panels.

Figure 3 (Panel A) shows the relationships of P/F plotted vs. Crs; for a decrease in Crs, P/F increased in patient S but decreased in NS. Panel B shows a decrease in P/F with increasing PEEP in patient NS; conversely (Panel C), P/F increased in S with decreasing PEEP.

**FIGURE 4:**
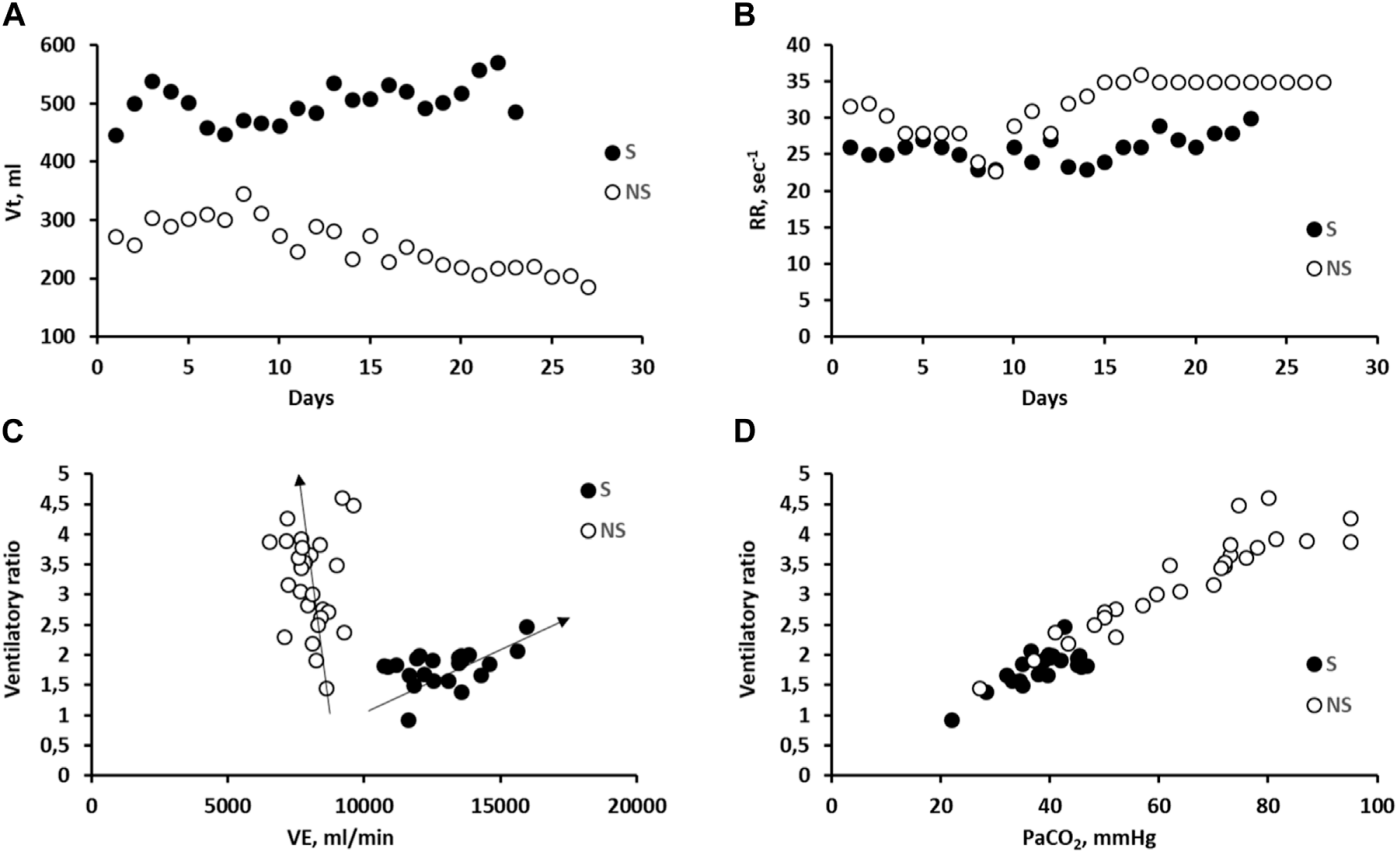
**(A)**: correlation between tidal volume (Vt) vs. days in subjects S and NS; **(B)**: correlation between respiratory rate (RR) vs. PEEP in subjects S and NS. **(C)**: correlation between day-time Ventilatory ratio vs. Ventilation (VE) in the two representative subjects S and NS. **(D)**: correlation between day-time Ventilatory ratio vs. PaCO_2_ in the two representative subjects S and NS.

Figure 4 shows the time course of tidal volume (Vt) and respiratory rate (RR) in S and NS patients (Panels A and B). By decreasing PEEP (Figure 1B), a higher driving pressure can increase Vt in S patient. Conversely, the opposite occurred in NS patient due to an increase in PEEP (Figure 1B), particularly considering the greater decrease in lung compliance (Figure 1A). Furthermore, it should be noted that in NS patients, the Vt approaches the anatomical dead space. The RR (Panel B) remained high for both subjects.

Concerning the ventilatory ratio, a clear dependence on ventilation is observed, albeit in opposite directions, considering S and NS patients (panel C). Panel D shows the striking dependence of the ventilatory ratio on PaCO_2_; in the case of patient S, PaCO2 remained within the physiological range, while it increased remarkably in patient NS.

## 4 Discussion

To our knowledge, this is the first study to compare the evolution of respiratory parameters in survivors and non-survivors mechanically ventilated SARS-CoV-2 patients hospitalised in ICU. This is a physiologic study and not a clinical study with the ambition of a validation phase of the results. The novelty is the longitudinal physiological data granularity, the stratification by outcome. The data interpretation is made on physiological models that offer a mechanistic reading to the ventilatory data and gas exchange behaviour in patients who did or did not survive at ICU discharge. We will discuss the differences defining a computational biophysical model allowing to define potential diffusion/perfusion limitations of alveolar gas exchanges.

**FIGURE 5:**
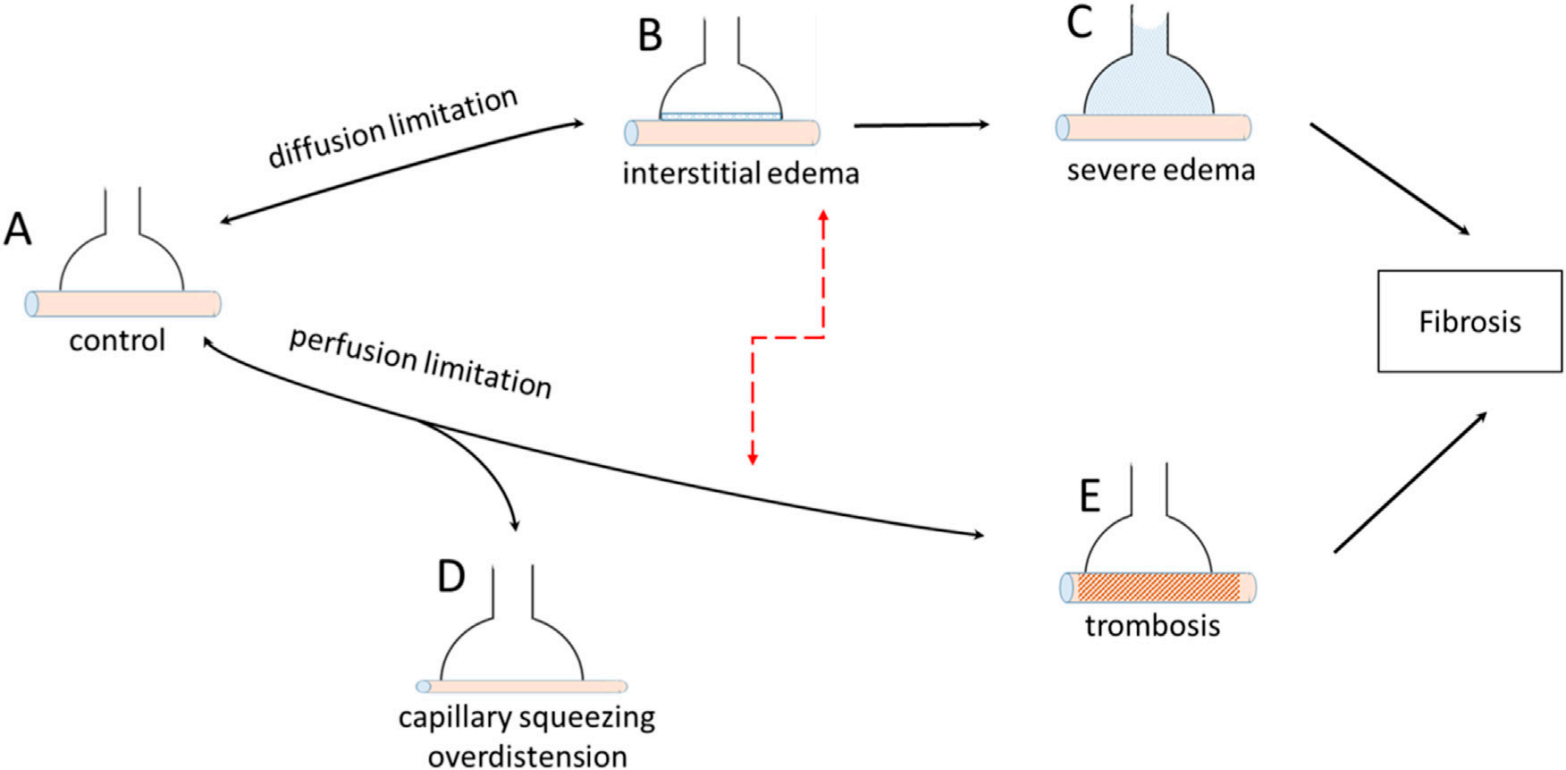
Various pathological conditions leading to diffusion limitation and perfusion limitation at the level of the air-blood barrier.

Figure 5 summarises the various conditions that may impact gas exchange at the alveolar level during the development of lung diseases, such as SARS-CoV-2 infection. Diffusion limitation may progress from a physiological condition (A) to interstitial oedema (B) and severe oedema with alveolar flooding (C). The development of oedema reflects an increase in microvascular permeability due to the progressive fragmentation of the proteoglycan component of the interstitial macromolecular network (Negrini et al., 1998; Negrini et al., 2008; Moriondo et al., 2012; Yi et al., 2016). The path from A to C indicates a progressive increase in the shunt effect. The path from A to E shows a case of perfusion limitation due to pulmonary capillary squeezing due to lung overdistension (D) or complete vessel closure due to thrombosis (E). The progression from A to D led to an increase in dead space. Red dashed arrows indicate mixed events that occur in severe lung pathology.

We interpret the decrease in *Crs* as mainly due to the loss of inflatable alveolar units (IAU) during disease progression and partly due to the increase in tissue elastance during the development of interstitial oedema (Dellacà et al., 2008). Gas exchange can occur only in the IAU, which retains its morphofunctional features to ensure gas diffusion.

### 4.1 Dependence of Vc on lung distension

**FIGURE 6:**
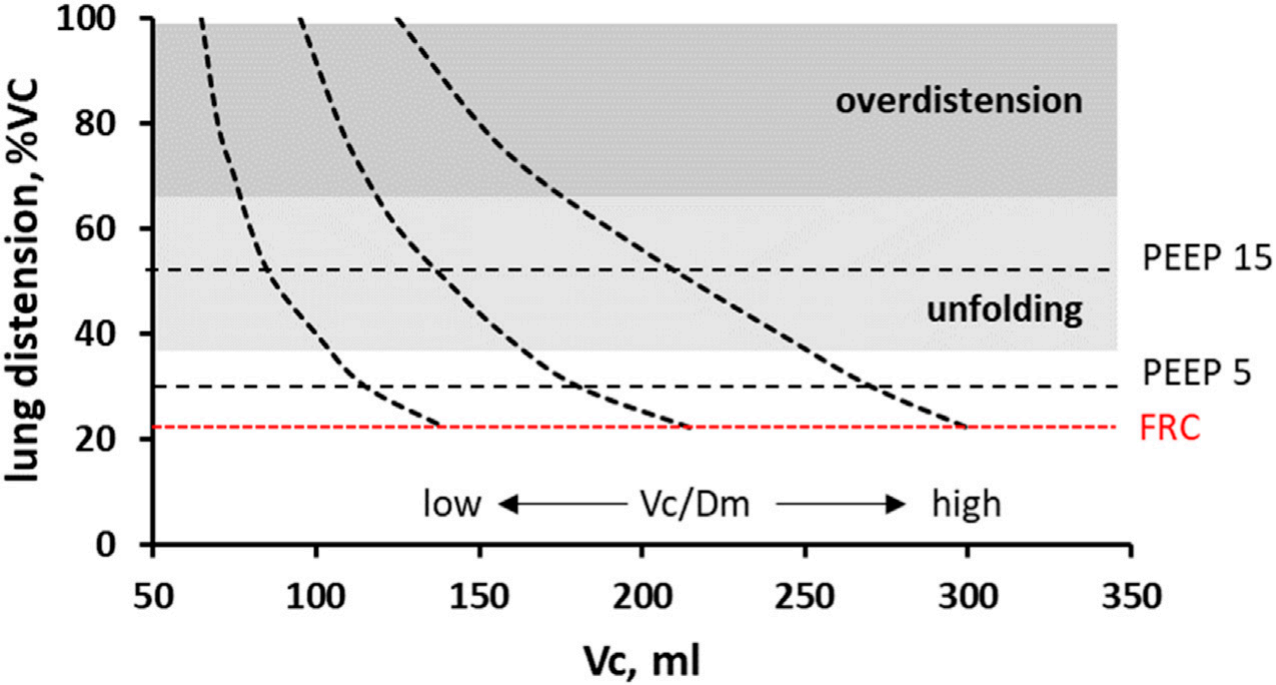
Decrease in capillary blood volume (Vc) on increasing lung distension, considering three cases of Vc values at Functional Residual Capacity (FRC) in the supine position. The Vc values correspond to either a low or high ratio to the diffusion capacity of the alveolar membrane (*Vc/Dm),* characterising inter-individual differences in alveolar morphology (from Miserocchi et al., 2008). Figure also refers to a PEEP of 5 and 10 cmH_2_O.

An increase in alveolar pressure leads to a decrease in capillary blood volume (*Vc*), owing to the squeezing of capillaries (Figure 5D) caused by an increase in parenchymal stretching (Glazier et al., 1969; Brower et al., 1985; Nieman et al., 1988; Koyama and Hildebrandt, 1991; Miserocchi et al., 2008). This decrease was found to vary remarkably among subjects, depending on the individual morphofunctional assembly of the alveolar capillary unit. The latter is characterised by the ratio of *Vc* to the diffusion capacity of the alveolar membrane (*Vc/Dm*), which essentially compares inter-individual differences in the extension of the pulmonary alveolar capillary network to the alveolar size (Miserocchi et al., 2008). The present available data do not allow for the estimation of inter-individual differences in *Vc/Dm* among patients. Accordingly, Figure 6 shows three cases of *Vc* ranging at Functional Residual Capacity (FRC) from 150 to 300 mL (corresponding to different *Vc/Dm* ratios). The figure reports the expected decrease in *Vc* with increasing lung distension with a PEEP of 5 and 15 cmH_2_O (Miserocchi et al., 2008): clearly, the decrease in *Vc* (in absolute terms) is larger the greater the *Vc* value at FRC (Figure 5D).

Interestingly, a decrease in pulmonary blood volume has been documented in post COVID-19 through Dual-energy CT scan not only in opacification areas but also in parenchyma of normal appearance in acute (Aydin et al., 2021; Ball et al., 2021) and post-acute phase (Mohamed et al., 2023) (Figure 5E), suggesting a potential limitation to perfusion.

### 4.2 Gas exchange

We have developed a model to estimate the dependence of alveolar gas exchange resulting from the functional coupling of blood capillary flow with gas diffusion flows (Beretta et al., 2019; Miserocchi et al., 2022; Miserocchi, 2023b). Our present aim is to rely on this model to compare two distinct conditions: hyperoxia and normocapnia in S patients, against hyperoxia and hypercapnia in NS patients. We shall briefly summarize the principles of the biophysical model.

Based on the gas mass conservation notion (Piiper and Scheid, 1981) and an exponential kinetics of the equilibration process, the alveolar-capillary equilibration for gas exchange reached at the exit of the blood from the capillary is mathematically defined as:

$$L_{eq}=e^{-\;\frac{Tt}{\tau }}$$ (1)

being *Tt* blood capillary transit time (also known as “*capillary residence time”* or “*blood contact time”*), and τ is the time constant of the exponential kinetics. *Tt* is the key parameter to switch from volumes to flows and can be estimated as the ratio of pulmonary blood capillary volume (*Vc*) to cardiac output ( $\dot{Q}$ ):

$$Tt=\frac{Vc}{\overset{´}{Q}}$$ (2)

The kinetics of the equilibration is defined by the time constant given by:

$$\tau =\frac{\beta Vc}{{DO}_{2}}\text{ for }O_{2}$$ (3)

and

$$\tau =\frac{\alpha Vc}{{DCO}_{2}}\text{ for }{\text{CO}}_{2}$$ (4)

being *DO*_*2*_ and *DCO*_*2*_, the respective diffusive capacities, while *β* and α include gas solubility and transport capacity in blood.

*Leq* can vary from 0 (perfect equilibration) to 1 (total lack of equilibration) (Beretta et al., 2019; Miserocchi et al., 2022; Miserocchi, 2023b).

This approach provided supplementary information to the classic ${\dot{V}}_{A}/\dot{Q}$ distribution (Wagner, 2008; Glenny and Robertson, 2011; Hopkins, 2020). Defining the kinetics of gas exchange equilibration that includes the estimate of the blood capillary transit time allows to develop the concept of “*shunt-like effect*” reflecting the decrease in $\frac{Vc}{\overset{´}{Q}}$ ratio. The latter was found to vary considerably among subjects, reflecting the heterogeneity (Miserocchi et al., 2008; Miserocchi, 2023b) of inborn morpho-functional arrangement of the air blood barrier as well as the individual response to functional conditions (lung stretching, hypoxia, increase in oxygen demand) (Miserocchi and Beretta, 2023).

### 4.3 Diffusion and perfusion limitation

The key issue on comparing S with NS is that both groups had a SatO_2_>90%; however, while the former remained normocapnic, the latter developed hypercapnia.

Diffusion limitation is a specific case occurring for oxygen, due to its low solubility-diffusion coefficients (Eq. 3).

**FIGURE 7:**
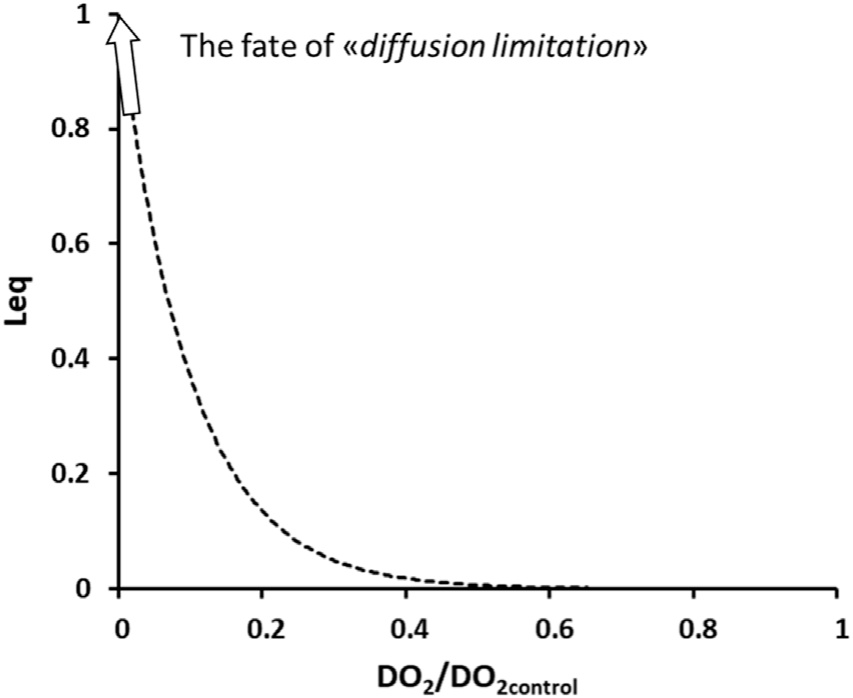
Exponential increase in *Leq* on decreasing diffusive capacitance for O_2_ relative to control.

Figure 7 shows that under physiological conditions, *Leq* = 0 at the exit of the pulmonary capillaries.

The development of interstitial and severe alveolar edema (Figures 5B, C) represent the obvious case of diffusion limitation for O_2_ uptake, due to the decrease in *DO*_*2*_. As shown in Figure 7, an exponential loss of equilibration capacity occurred with a 5-times decrease in *DO*_*2*_, compatible with the observed average decrease in *Crs* in patients relative to a physiological value of approximately 100 mL/cmH_2_O. The loss of O_2_ equilibration capacity for the whole lung simulates a “*shunt-like effect.*”

Oxygen diffusion limitation is commonly compensated for by an increase in F_I_O_2_. One shall report that for FIO_2_ > 0.7 (Aggarwal et al., 2018), cellular (Kistler et al., 1967; Weibel, 1971) and tissue (Chow et al., 2003; Kallet and Matthay, 2013) damage in the lungs were reported, leading to increased alveolar permeability (Matalon and Egan, 1981; Kolliputi et al., 2010).

Conversely, a diffusion limitation is hardly conceivable for CO_2_ exchange considering its high solubility-diffusion coefficients. Accordingly, one can develop the hypothesis of perfusion limitation.

**FIGURE 8:**
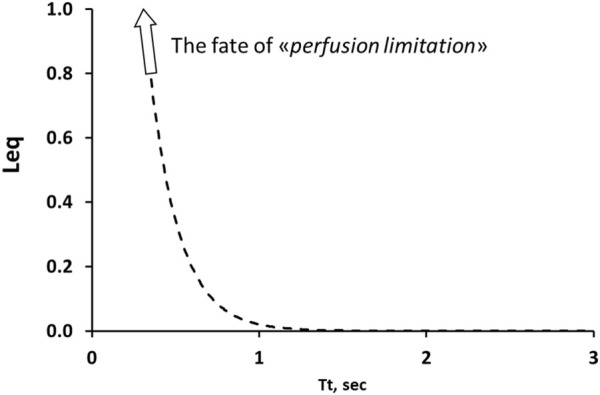
Computational model for cause-effect interpretation of perfusion limitation in CO_2_ removal. Perfusion limitation expressed by the increase in Leq on decreasing Tt. Note the exponential increase in Leq for Tt < 1 s.

The aim of our study is to find a cause-effect relationship for developing hypercapnia. Figure 8 presents a computational (Eq. 1) estimate of the exponential increase in perfusion limitation for CO_2_ removal for *Tt* < 1 s by decreasing *Vc* (Eq. 2) (Figures 5D, E).

Besides a decrease in Vc due to pulmonary stretching, a further factor arises from tissue compression in developing oedema (Figures 5B, C), that could actually lead to complete vessel closure (Mazzuca et al., 2019), functionally equivalent to the case of thrombosis (Figure 5E). Furthermore, studies on ECMO have confirmed that CO_2_ removal is hampered by low blood flow (Karagiannidis et al., 2017; Giraud et al., 2021; Zanella et al., 2022).

### 4.4 Lung fluid balance

**FIGURE 9:**
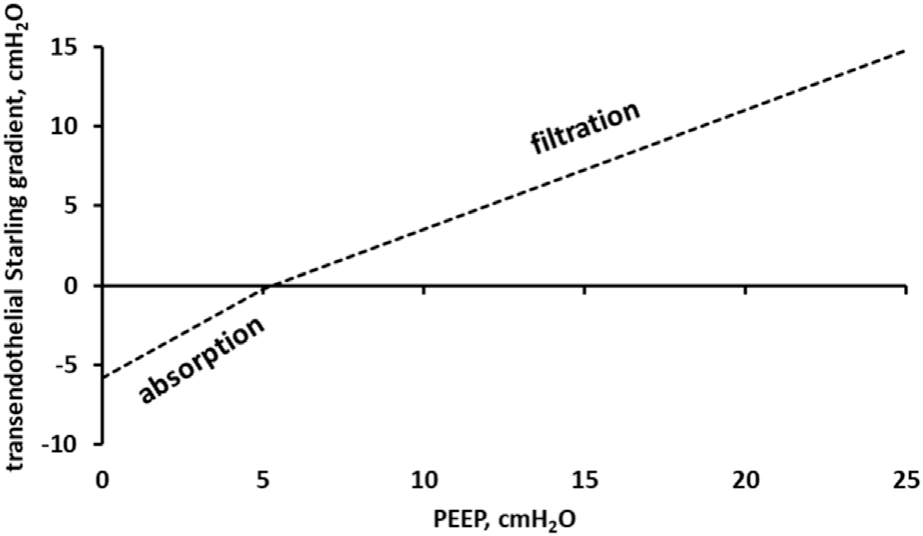
Starling pressure gradient across the lung capillary endothelium as a function of PEEP.

An estimate of the Starling pressure gradient controlling the lung fluid balance, particularly alveolar pressure, is presented in Figure 9. We accounted for the hydraulic and colloidal osmotic pressure, reflection coefficient, and alveolar surface tension (Beretta et al., 2021).

Data in Figure 9 show that, for Palv>5 cmH_2_O, the transendothelial Starling gradient favours microvascular filtration and thus is an edemagenic factor (Miserocchi et al., 1993).

Lung overdistension has been found to increase microvascular filtration (Miserocchi et al., 1991), a finding confirmed by a computational model showing stress-dependent leak progression through an epithelial monolayer (Hamlington et al., 2016; Hamlington et al., 2018).

Notably, a decrease in Tt, resulting in an increase in blood velocity, leads to an increase in shear rate (Miserocchi et al., 2022), which in turn causes the increase in microvascular and protein permeability, thus favouring oedema (Sill et al., 1995; Lakshminarayanan et al., 2000; Mazzag et al., 2003; Barakat et al., 2006; Kang et al., 2014; Kolářová et al., 2014; Miserocchi and Beretta, 2023).

### 4.5 Study limitations

We have to acknowledge some study limitation. We did not have any specific exclusion criteria. However, we did not have information on the screening data of ICU admission, but this is a convenient sample size of patients admitted to ICU of the Arequipa Hospital in Perù with a clinical diagnosis of respiratory failure with a positive PCR confirmation of Sars-CoV2 infection that were enrolled from April 2020 to March 2021. This study is not designed to evaluate independent association of clinical variables on outcome by using multivariable models of association but aims at exploring the physiopathology of gas exchange in the air-blood barrier during acute respiratory failure. We relied on established computational physio-pathological models.

### 4.6 Concluding remarks

This paper deals with diffusion and perfusion limitation to alveolar gas exchanges in mechanically ventilated COVID-19 patients.

In the representative NS patient, P/F decreased in the first week of ICU stay (Figure 2A), clearly reflecting oxygen diffusion limitation compensated by an increase in PEEP and FIO_2_ (Figures 1B, 2C, respectively). This was in accordance to the PEEP:FIO_2_ tables (Brower et al., 2004) and the guidelines (Fan et al., 2017), the leading idea being to increase alveolar recruitment and ventilation to favor oxygen uptake, although a steady SatO_2_>90%. However, the increase in PEEP may contribute to a progressive increase in PaCO_2_ (Reazoagli and Bellani, 2022), due to perfusion limitation, hindering CO_2_ removal (Figure 2E) and leading to a remarkable increase in the ventilatory ratio – which -in turn – is a marker *per se* of the respiratory failure severity (Figure 4D).

In representative S patient, the increase in P/F over the first week (Figure 2A) led to the decision (Brower et al., 2004) to decrease both PEEP and FIO_2_ (Figures 1B, 2C). Consequently, this allowed ventilation of patient S with a progressively lower mean airway pressure, favoring CO_2_ removal, notwithstanding the potential for alveolar de-recruitment.

In NS group, the ventilatory strategy led to SatO_2_ >90% coupled with severe hypercapnia. The latter has been considered as a biomarker of increased dead space due to perfusion limitation, a condition associated independently with a high risk of mortality (Nuckton et al., 2002).

In fact, survivors or non-survivors are separated by a faint border considering that in both groups, lung compliance was decreased by the disease to approximately 1/5 of normal, meaning that the total number of alveolar units assuring gas exchange was decreased from a physiological value of approximately 500 (Ochs et al., 2004) to 100 million. One cannot exclude an overestimate of the decrease in *Crs* in the study population considering the possible presence of auto-PEEP, which may occur with an increasing respiratory rate (Marini, 2011). In severe cases of mechanically ventilated patients, a 70%–80% reduction in DLCO relative to the expected normal value was reported at 5–12 months (Krueger et al., 2023; van Willigen et al., 2023). Radiological pulmonary abnormalities have been described more than 100 days after the diagnosis of COVID-19 (Sonnweber et al., 2021). It appears reasonable to relate these decreased variables to pulmonary fibrosis development (Figure 5).

Several parameters obviously impact on the efficiency of gas exchanges in the air blood barrier.

The time dependent analysis that we performed allows an integrated view coherent with the parameters considered, highlighting the time dependence of gas exchanges in the air blood barrier, being diffusion limited for O_2_ and perfusion limited for CO_2_.

## Data Availability

The raw data supporting the conclusions of this article will be made available by the authors, without undue reservation.
